# Using the Goal Attainment Scale adapted for depression to better understand treatment outcomes in patients with major depressive disorder switching to vortioxetine: a phase 4, single-arm, open-label, multicenter study

**DOI:** 10.1186/s12888-021-03608-1

**Published:** 2021-12-11

**Authors:** Maggie McCue, Sara Sarkey, Anna Eramo, Clement François, Sagar V. Parikh

**Affiliations:** 1 Takeda Pharmaceuticals U.S.A., Inc., 95 Hayden Avenue, Lexington, MA 02421 USA; 2 Lundbeck LLC, 6 Parkway North Blvd, Deerfield, IL 60015 USA; 3 University of Michigan Health, 1500 E. Medical Center Dr, Ann Arbor, MI 48109 USA

**Keywords:** Major depressive disorder, Vortioxetine, Goal attainment, Goal achievement

## Abstract

**Background:**

Major depressive disorder (MDD) is the leading cause of disability worldwide. Response to pharmacologic treatment is generally evaluated by traditional clinician- and patient-reported rating scales. Assessing therapeutic efficacy using the Goal Attainment Scale offers a complementary measure that focuses on recovery-oriented outcomes that patients consider valuable and vital to their well-being. This study aimed to examine outcomes using the Goal Attainment Scale adapted for depression (GAS-D).

**Methods:**

A phase 4, single-arm, open-label, multicenter study enrolled patients with MDD who were switching antidepressant medication. Patients received vortioxetine 10–20 mg over 12 weeks. Three specific, measurable, attainable, relevant, and time-bound goals were collaboratively set by patients with their clinicians. One goal was determined by the patient’s self-defined objectives; 2 were related to predefined domain categories. Prespecified domains included psychological, motivational, emotional, physical/functional, and cognitive categories. The primary endpoint was the proportion of patients who achieved a GAS-D score ≥ 50 at week 12. Secondary and exploratory endpoints included changes from baseline in several clinical and patient-reported measures of depression and cognitive function. Safety and tolerability were also assessed.

**Results:**

At week 12, of the 122 adults participating in the study, 57.8% achieved a GAS-D score ≥ 50. Depression severity, cognitive function, cognitive performance, well-being, employment, and quality of life also significantly improved. Treatment response and remission rates were approximately 65 and 40%, respectively. Vortioxetine was well tolerated, with adverse events consistent with product labeling.

**Conclusions:**

A majority of patients with MDD switching to vortioxetine achieved their treatment goals, including improvement in specific functional outcomes relating to physical and emotional goals, as assessed by the GAS-D and standard patient- and clinician-reported measures. When assayed for convergent validity in a separate analysis, changes in goal scores on the GAS-D were statistically significantly correlated with multiple commonly used clinical measures of depression assessed in this study. The GAS-D approach provides a new patient-centric paradigm for the collaborative development and assessment of progress toward meaningful treatment goals, contributing to a comprehensive evaluation of treatment outcomes in patients with MDD. Longer studies against a control intervention are justified.

**Trial registration:**

ClinicalTrials.gov identifier: NCT02972632. Registered 21 November 2016.

**Graphical Abstract:**

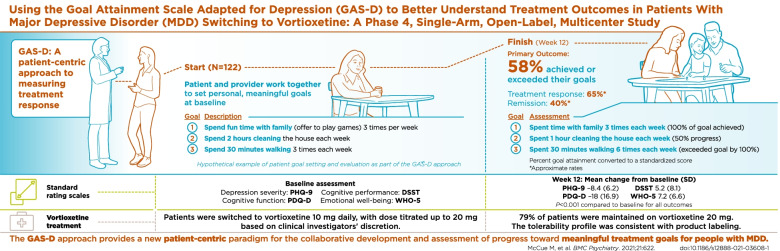

**Supplementary Information:**

The online version contains supplementary material available at 10.1186/s12888-021-03608-1.

## Background

Major depressive disorder (MDD) is the leading cause of disability worldwide, affecting ~8.3% of the population in the United States [1–3]. MDD accounts for 4.3% of the global burden of disease, and its costs in the United States alone total $99 billion annually [3, 4]. Accordingly, MDD is associated with substantial economic and social costs.

Response to treatment for MDD is generally evaluated by traditional clinician- and patient-reported rating scales [5, 6]. These scales focus on symptoms, however, and do not address meaningful changes specific to an individual patient’s condition. A complementary approach that focuses on response to treatment in an individualized manner is one that uses the Goal Attainment Scale (GAS), developed by Kiresuk and Sherman in 1968 [7]. This scale uses a semiquantitative approach incorporating a patient’s individual expectations from treatment by assessing outcomes against specific, measurable, attainable, relevant, and time-bound (SMART) goals [7, 8], allowing for acceptable inter-rater reliability [9–11]. Outcomes assessed using GAS complement traditional clinical scales; the GAS has demonstrated utility in measuring progress in recovery-oriented outcomes that patients consider valuable and vital to their well-being in both medical and nonmedical indications that are otherwise difficult to assess [8, 12, 13]. Furthermore, progress toward multiple goals can be converted into a standardized *T* score, facilitating comparisons between individual patients with different goals and between treatment modalities [7].

The GAS approach has been shown to be a useful method for assessing outcomes for patients undergoing physical rehabilitation [14, 15] and patients with mental health conditions [12]. This approach may therefore be particularly appealing for use in patients with MDD.

Goal setting can help patients progress toward desired treatment outcomes relating to behavioral changes and can also improve engagement with healthcare providers [16–18]. Goal setting and defining treatment success as goal achievement are integral to cognitive behavioral therapy (CBT) and to the application of the GAS adapted for depression (GAS-D) approach for patients with MDD. Both approaches involve identifying a behavior associated with the symptoms of MDD and setting goals representing a desirable behavior change. As part of the CBT process, these goals are regularly revisited, barriers to progress challenged, and goal achievement reinforced to drive progress [19, 20]. In contrast, goal setting using the GAS-D approach occurs before initiating therapy, and progress against these goals is assessed without active follow-up or reinforcement, offering a novel method of investigating the efficacy of pharmacologic therapies.

Accordingly, the feasibility of applying the GAS approach in patients with MDD was evaluated in a recent study of patient attitudes toward setting treatment goals in MDD, and the authors reported that patients see value in this approach because it affords patients the opportunity to provide input into the design of their treatment plans, while setting a framework against which progress can be assessed [21]. Further, as described in a recent commentary, symptoms of depression, particularly cognitive and physical symptoms, are heterogeneous; therefore, using an individualized measure to assess response to treatment may encourage patients to focus on symptoms, functional improvements, and goals that resonate most with them, and to work toward integrating their goals into their daily lives [22].

Collaborative goal setting is also encouraged when managing patients with MDD, but current measures of treatment success applied in clinical studies of patients with MDD overlook goal achievement as a primary endpoint in favor of classic symptom rating scales. To address the disconnect between clinical research and clinical practice, the GAS-D was developed to provide a scoring system aligned with outcome assessment in real-world clinical practice [22, 23].

The present study implemented the GAS-D as the primary outcome measure to examine its effectiveness in evaluating outcomes of a 12-week course of treatment with the antidepressant vortioxetine (Trintellix, Takeda Pharmaceuticals America, Inc.; Lexington, MA, USA). To our knowledge, this is the first study conducted to evaluate treatment for MDD that uses the goal attainment approach as a primary outcome measure. The GAS-D was used to assess progress toward predetermined personalized treatment goals that patients set in collaboration with their clinicians in tandem with standard measures of antidepressant efficacy relating to depressive symptoms, clinical global impression, cognitive functioning, and well-being. The study was also designed to determine treatment response and remission rates and the safety and tolerability of vortioxetine in patients with MDD.

## Methods

### Study design

This phase 4, single-arm, open-label, multicenter clinical trial conducted in the United States (ClinicalTrials.gov ID NCT02972632) evaluated the effectiveness of a 12-week course of vortioxetine treatment on goal achievement. Patients with MDD between ages 18–65 years who were recently or currently receiving treatment with an approved antidepressant for ≥6 weeks and were considered to be appropriate for a change in medication were eligible for inclusion in the study. Patients were also required to have a Patient Health Questionnaire-Depressive Symptoms (PHQ-9) score ≥ 5 and a Clinician Global Impression-Severity (CGI-S) score ≥ 4 at screening. Excluded from the study were patients diagnosed with a current psychiatric disorder other than MDD (except non-primary concurrent anxiety), and those who were considered to be at imminent risk for hospitalization due to severe depression or who posed a significant risk for suicide.

All patients provided written informed consent prior to their participation. The study was conducted in accordance with the Declaration of Helsinki and the International Council for Harmonisation of Technical Requirements for Pharmaceuticals for Human Use (ICH) guidelines. The study protocol was approved by institutional review boards at all participating sites.

### Procedures

Patients were initiated on vortioxetine 10 mg and titrated to 20 mg over the treatment period based on the clinical investigators’ discretion. Follow-up occurred at weeks 2, 4, 6, 9, and 12. Follow-up interviews at weeks 4 and 9 were conducted by telephone, and all other follow-up interviews were conducted in person. A safety follow-up telephone interview took place at week 16.

**Fig. 1:**
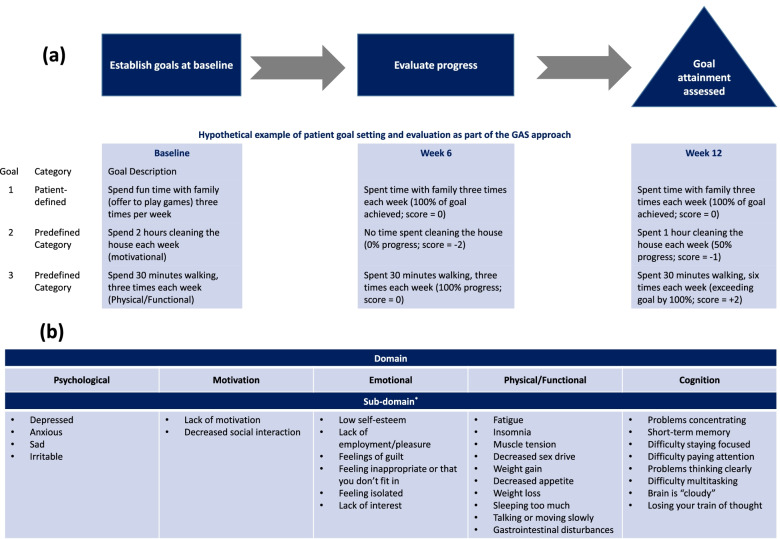
Goal-setting and domain-defined approach. **a** Establishing and assessing progress toward treatment goals. **b** Goal domains. *Not a comprehensive list. GAS, Goal Attainment Scale

Treatment goals were collaboratively set by the patient and clinician using the GAS-D approach at baseline and were not revisited or reinforced during the course of the study (Fig. 1A and B). Three treatment goals were set: 1 determined by the patient’s self-defined objectives and 2 related to predefined domain categories. Prespecified domains included psychological, motivational, emotional, physical/functional, and cognitive categories, each with multiple subdomains. The goals were SMART [8]; for example, “spend fun time with family (offer to play games) 3 times per week” is a SMART goal aimed at increasing enjoyment and pleasure.

Each goal outcome was assigned a score: −2 = baseline performance; −1 = 50% progress toward goal; 0 = goal/target outcome achieved; 1 = 50% better than goal; 2 = 100% better than goal. The composite GAS-D score was then transformed to a standardized *T* score using the formula found in Supplementary Fig. 1, with a *T* score = 50 indicating that all goals were achieved as expected, <50 indicating goals were achieved less than expected, and > 50 indicating goals were achieved better than expected (see Supplementary Fig. 1, Additional file 1).

### Endpoints

The primary endpoint was an estimation of the proportion of patients who achieved their goals as represented by an overall GAS-D score ≥ 50 at week 12. Change from baseline in mean GAS-D score was also assessed at weeks 6 and 12 as a secondary endpoint. Additional secondary endpoints included change from baseline at weeks 6 and 12 in measures of depression severity and response to treatment (PHQ-9, Clinician Global Impression-Improvement [CGI-I]), cognitive function (Perceived Deficits Questionnaire-Depression [PDQ-D]), emotional well-being (World Health Organization-Five Well-Being Index [WHO-5]), and QoL (Quality of Life Enjoyment and Satisfaction Questionnaire [Q-LES-Q]). PHQ-9 scores ≤4 were considered to represent minimal symptoms that may not require treatment, CGI-I scores ≤2 represented much improvement, and CGI-S scores ≤2 represented remission. Exploratory endpoints included the Lam Employment Absence and Productivity Scale (LEAPS), the Virtual Reality Functional Capacity Assessment Tool (VRFCAT), and the Digit Symbol Substitution Test (DSST). Safety and tolerability measures (adverse events [AEs], AEs leading to discontinuation, changes in weight, and the Columbia-Suicide Severity Rating Scale [C-SSRS]) were also assessed.

### GAS-D validity

The GAS-D was assayed for convergent validity in a post hoc analysis of data from the present study, and changes in goal scores on the GAS-D were found to have a statistically significant correlation with several commonly used clinical measures of depression (see Supplementary Tables 1–3, Additional file 2) [23].

### Statistical analyses

The estimated proportion and 95% confidence interval were calculated for patients who achieved goals as demonstrated by a GAS-D score ≥ 50 at week 12 using SAS software version 9.4. Paired *t*-tests were performed to calculate changes from baseline, along with *P* values for efficacy variables. Two-sided *P* < 0.05 was considered statistically significant.

## Results

### Baseline characteristics

This study enrolled 122 patients. Baseline patient characteristics and disposition are presented in Table 1.

**Table 1:** Baseline patient characteristics (safety analysis set; N = 122)

**Sex, n (%)**	
Male	21 (17.2)
Female	101 (82.8)
**Age, mean years (SD)**	45.3 (12.2)
≤55 years, n (%)	95 (77.9)
**Race, n (%)**	
White	83 (69.2)
Black or African American	28 (23.3)
Asian	5 (4.2)
Native Hawaiian or Other Pacific Islander	1 (0.8)
Multiracial^a^	3 (2.5)
Unknown	2 (1.6)
**Ethnicity, n (%)**	
Hispanic or Latino	28 (23.0)
**Employment status, n (%)**^**b**^	
Employed full-time	32 (27.6)
Employed part-time	22 (19.0)
Self-employed	15 (12.9)
Not employed^c^	48 (41.7)
**BMI, mean kg/m**^**2**^ **(SD)**	34.1 (9.7)
**PHQ-9,**^**d**^ **mean (SD)**	15.7 (4.8)
**Previous antidepressant medication, n of medications (%)**	
**SSRIs**	
Fluoxetine	34 (16.8)
Sertraline	25 (12.4)
Escitalopram	24 (11.9)
Citalopram	25 (12.4)
Paroxetine	11 (5.4)
**SNRIs**	
Venlafaxine	21 (10.4)
Duloxetine	9 (4.5)
Desvenlafaxine	3 (1.5)
**NDRIs**	
Bupropion	32 (15.8)
**Other**	
Trazodone	9 (4.5)
Vilazodone	6 (3.0)
Amitriptyline	1 (0.5)
Doxepin	1 (0.5)
Mirtazapine	1 (0.5)
**Reason for switching from previous medication, n (%)**	
Inadequate response	39 (32)
Not working fast enough	37 (30)
Not meeting treatment goals	28 (23)
Problems with focus/concentration	9 (7)
Side effect	5 (4)
Other	5 (4)
**Patients with concurrent medical conditions, n (%)**	122 (100)
Blood and lymphatic system disorders	4 (3.3)
Cardiac disorders	1 (0.8)
Congenital, familial, and genetic disorders	2 (1.6)
Ear and labyrinth disorders	3 (2.5)
Endocrine disorders	20 (16.4)
Eye disorders	17 (13.9)
Gastrointestinal disorders	29 (23.8)
General disorders	4 (3.3)
Hepatobiliary disorders	2 (1.6)
Immune system disorders	41 (33.6)
Infections and infestations	13 (10.7)
Investigations	17 (13.9)
Metabolism and nutrition disorders	46 (37.7)
Musculoskeletal and connective tissue disorders	41 (33.6)
Neoplasms benign, malignant, and unspecified (including cysts and polyps)	1 (0.8)
Nervous system disorders	44 (36.1)
Renal and urinary disorders	6 (4.9)
Reproductive system and breast disorders	10 (8.2)
Respiratory, thoracic, and mediastinal disorders	26 (21.3)
Skin and subcutaneous tissue disorders	17 (13.9)
Social circumstances	23 (18.9)
Surgical and medical procedures	4 (3.3)
Vascular disorders	27 (22.1)
**Patients with concurrent psychiatric disorders,**^**e**^ **n (%)**	
Insomnia related to another medical condition	28 (23.0)
Anxiety	18 (14.8)
Depression	17 (13.9)
Generalized anxiety disorder	5 (4.1)
Insomnia	5 (4.1)
Attention deficit/hyperactivity disorder	2 (1.6)
Initial insomnia	1 (0.8)
Nightmare	1 (0.8)
Obsessive compulsive disorder	1 (0.8)
Panic disorder	1 (0.8)
Performance fear	1 (0.8)
Post-traumatic stress disorder	1 (0.8)
Sleep disorder	1 (0.8)
Stress	1 (0.8)

Seventeen (13.8%) patients withdrew from the study: 10 voluntarily withdrew, 4 were lost to follow-up, and 3 discontinued following an adverse event

^a^Patient checked more than one race option on the case report form. ^b^n = 115. In cases of multiple employment statuses per patient, the patient is counted as many times as the number of employment statuses. Percentages are based on the number of patients in the full analysis set. Data for 1 patient were missing. ^c^Includes unemployed, student, retired, nonworking spouse, and other. ^d^n = 110. ^e^Psychiatric disorders excluding major depressive disorder

*BMI* body mass index, *NDRI* norepinephrine-dopamine reuptake inhibitor, *PHQ-9* Patient Health Questionnaire-Depressive Symptoms, *SD* standard deviation, *SNRI* serotonin and norepinephrine reuptake inhibitor, *SSRI* selective serotonin reuptake inhibitor

Overall, study participants were predominantly white (69.2%), female (82.8%), and ≤55 years of age (77.9%). Mean PHQ-9 score was 15.7 (moderately severe depression), and 58.3% of the patients were employed. All 122 patients had comorbid medical conditions, including comorbid psychiatric conditions, insomnia related to another condition (23.0%), and anxiety (14.8%), which are consistent with what may be expected in a phase 4 effectiveness study population that has few exclusion criteria. Approximately 79% of patients were successfully titrated to 20 mg vortioxetine and remained on that dose for a portion of the study period; 104 patients were treated for ≥11 weeks.

Of the previously prescribed antidepressant treatments, selective serotonin reuptake inhibitors were the most frequently prescribed class of antidepressant. Patients expressed several reasons for switching medication, with the predominant focus on lack of efficacy. The most common reasons for switching included inadequate response to a previous antidepressant (32%); a previous antidepressant not working fast enough (30%); and an inadequate treatment response expressed by failing to meet goals (23%).

### Patients achieving GAS-D score ≥ 50 (primary endpoint)

**Fig. 2:**
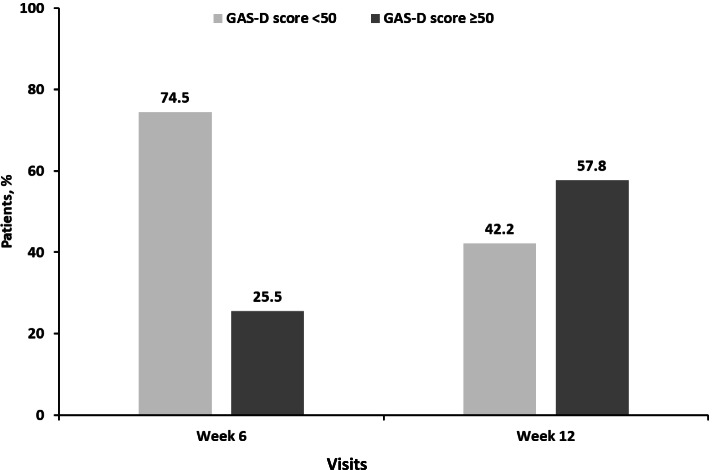
Percentage of patients with GAS-D scores <50 (goals achieved less than expected overall) and ≥ 50 (all goals achieved or exceeded overall; primary endpoint) at weeks 6 and 12 of treatment^a^. 95% CI (17.3–33.6) and (48.5–67.1) at weeks 6 and 12, respectively, for patients with a GAS-D score ≥ 50. n = 109 (week 12). ^a^Each goal outcome was assigned a score: −2 = baseline performance; −1 = 50% progress toward goal; 0 = goal/target outcome achieved; 1 = 50% better than goal; 2 = 100% better than goal. The overall GAS-D score was calculated based on standardizing the score to a central value of 50 (see **Supplementary Fig. 1,** Additional File 1). CI, confidence interval; GAS-D, Goal Attainment Scale adapted for depression

**Fig. 3:**
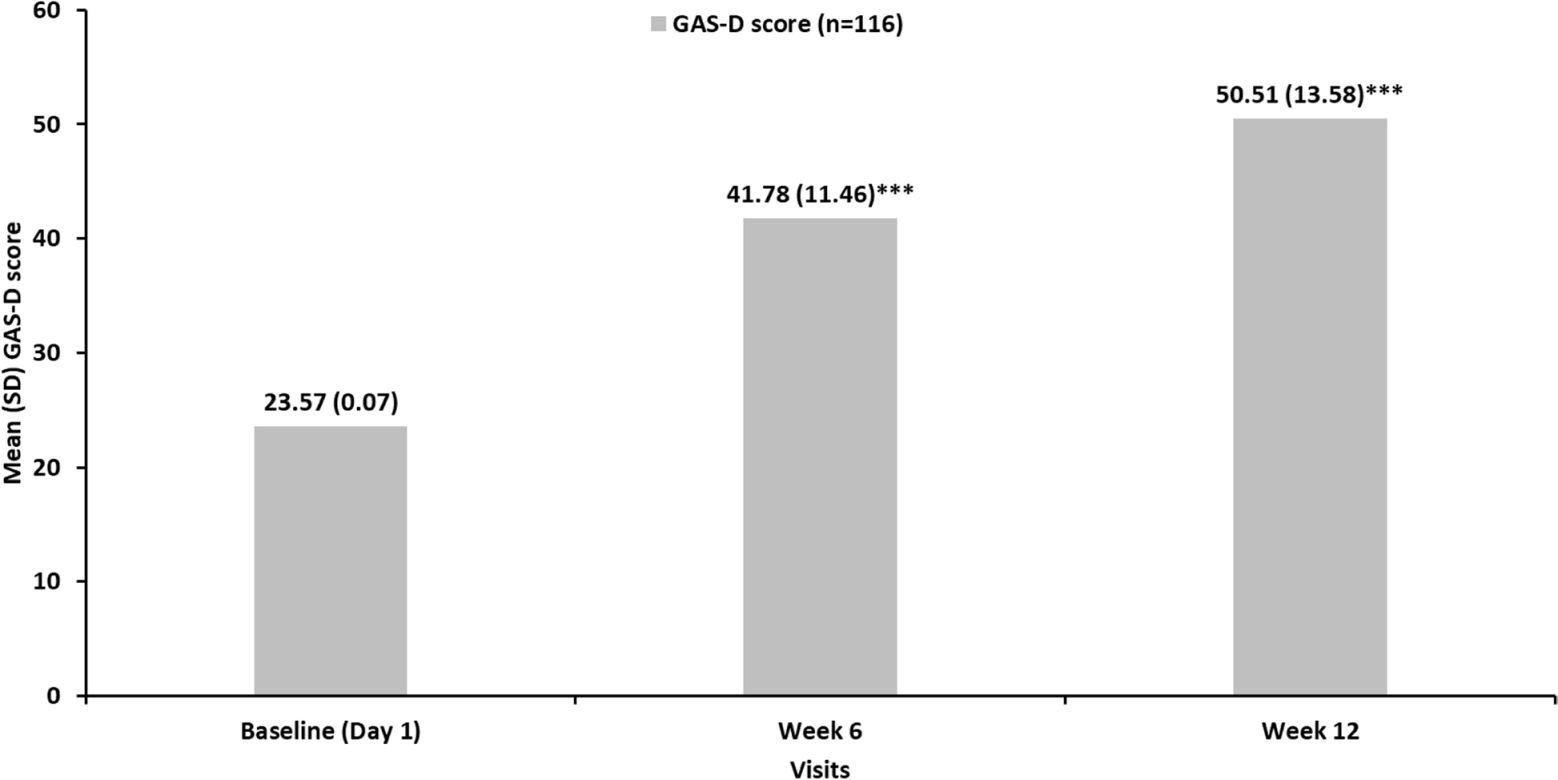
Mean standardized GAS-D scores by visit^a^. ***P < 0.001 for change from baseline using paired *t*-tests. ^a^Each goal outcome was assigned a score: −2 = baseline performance; −1 = 50% progress toward goal; 0 = goal/target outcome achieved; 1 = 50% better than goal; 2 = 100% better than goal. The overall GAS-D score was calculated based on standardizing the score to a central value of 50 (see **Supplementary Fig. 1,** Additional File 1). A standardized GAS-D score ≥ 50 represents overall goal achievement. n = 109 (week 12). GAS-D, Goal Attainment Scale adapted for depression; SD, standard deviation

At week 12 of treatment, 57.8% of patients achieved a GAS-D score ≥ 50 (all goals achieved or exceeded overall). Significant changes in GAS-D score versus baseline were observed at weeks 6 and 12 (Figs. 2 and 3). The highest number of goals were set in the motivational and physical/functional goal domains (31 and 32%), while the highest percent of goals achieved occurred within the emotional domain. The most common goal subdomains included lack of motivation, fatigue, insomnia, lack of enjoyment/pleasure, feeling isolated, depressed mood, and problem concentrating.

### Depressive symptoms and functional outcomes (secondary endpoints)

In addition to GAS-D assessment of goal achievement during treatment with vortioxetine, measures of depression severity (PHQ-9), cognitive function (PDQ-D), cognitive performance (DSST), and emotional well-being (WHO-5) showed statistically significant improvements from baseline at weeks 6 and 12 (Table 2). Response to treatment, as indicated by a ≥ 50% reduction in total PHQ-9 score and a CGI-I score ≤ 2 indicating “much improvement,” was reported by 64.2 and 65.8% of patients, respectively. Moreover, at week 12, 38.7% of patients presented with minimal symptoms that may not require treatment (PHQ-9 ≤ 4) and 44.1% of patients met the definition of remission (CGI-S ≤ 2).

**Table 2:** Measures of depression severity (PHQ-9), cognitive function (PDQ-D), cognitive performance (DSST), and emotional well-being (WHO-5)

Measures	Baseline (Day 1)	Week 6	Week 12
**PHQ-9**			
n	110	109	111
Mean (SD)	15.7 (4.8)	8.9 (5.9)	7.1 (5.6)
**Change from baseline**			
n	–	103	106
Mean (SD)	–	−6.5 (6.2)	−8.4 (6.2)
*P* value	–	<0.001	<0.001
**PDQ-D**			
n	110	106	108
Mean (SD)	40.5 (14.9)	25.7 (14.9)	22.2 (15.9)
**Change from baseline**			
n	–	104	106
Mean (SD)	–	−14.8 (13.7)	−18.0 (16.9)
*P* value	–	<0.001	<0.001
**DSST**			
n	113	107	107
Mean (SD)	46.7 (11.1)	50.6 (12.9)	52.1 (11.7)
**Change from baseline**			
n	–	105	105
Mean (SD)	–	3.8 (9.8)	5.2 (8.1)
*P* value	–	<0.001	<0.001
**WHO-5**			
n	115	109	109
Mean (SD)	5.6 (3.48)	10.8 (5.5)	12.8 (6.2)
**Change from baseline**			
n	–	108	109
Mean (SD)	–	5.1 (5.4)	7.2 (6.6)
*P* value	–	<0.001	<0.001

*DSST* Digit Symbol Substitution Test, *PDQ-D* Perceived Deficits Questionnaire-Depression, *PHQ-9* Patient Health Questionnaire-Depressive Symptoms, *SD* standard deviation, *WHO-5* World Health Organization-Five Well-Being Index

There was a significant overall improvement at week 12 on the Q-LES-Q (see Supplementary Fig. 2, Additional file 1) and a significant improvement on LEAPS (see Supplementary Fig. 3, Additional file 1). There was no significant difference in total time (mean change from baseline: –17.66 s), errors (0), or forced progressions (−0.1) on the VRFCAT at week 12.

### Safety and tolerability

The safety profile of vortioxetine during this study was consistent with the vortioxetine product labeling [24]. Overall, 117 AEs deemed related to study treatment were reported by 59 patients. AEs were most commonly gastrointestinal (34%), nervous system (21%), or psychiatric (20%) disorders and were generally mild or moderate in severity.

Other AEs reported by ≥5% of patients included nausea, headache, anxiety, constipation, and diarrhea (Table 3). A total of 7 AEs in 6 patients led to treatment discontinuation. These AEs included 4 cases of psychiatric disorders and 1 case each of headache, nausea, and vomiting. In 1 patient, 2 serious treatment-emergent AEs—depression and suicidal ideation—were reported, leading to treatment discontinuation. These events were determined to be unrelated to the study drug. No suicides or deaths occurred during the study. Patients treated with vortioxetine experienced a 0.6 kg mean increase in weight from baseline.

**Table 3:** Adverse events reported by ≥5% of patients (N = 122)

Adverse event	n (%)
Patients with any TEAE	83 (68.0)
Nausea	26 (21.3)
Headache	11 (9.0)
Anxiety	8 (6.6)
Constipation	7 (5.7)
Diarrhea	7 (5.7)

*TEAE* treatment-emergent adverse event

## Discussion

Patient-centric medicine remains a key objective for society and includes the concept of achieving outcomes that are explicitly relevant to each individual. The goal attainment approach using the GAS-D provides the framework for a collaborative conversation between the patient and their clinician, serving to align them on treatment goals through shared decision making, while prioritizing what matters most to the patient. This patient-centric approach offers a unique method of assessing response to treatment in patients with MDD. Applying a goal attainment approach also provides a more holistic assessment of the effect of antidepressant therapy than conventional scales alone.

In this study, the GAS-D was employed as the primary outcome measure to assess the performance of an antidepressant in treating depression. Specifically, we found that a majority of patients with MDD who required a switch in antidepressant medication to vortioxetine achieved their treatment goals. In addition, there were improvements in more traditional measures of depressive symptoms. This phase 4, open-label study also demonstrated the overall effectiveness of vortioxetine, given that the patient sample in this study was representative of real-world patients, with varying medication histories, comorbidities, and levels of functioning, and who had experienced treatment failure with other antidepressants. Vortioxetine was also well tolerated, with its safety profile reflecting its current product labeling [24].

In this study, patients with MDD were switched to vortioxetine, a multimodal antidepressant that has a demonstrated ability to alleviate mood and physical and cognitive symptoms as assessed using conventional scales [24–26]. The high proportion of patients who achieved a GAS-D score ≥ 50, paralleling clinical improvements on several standard patient- and clinician-reported measures, indicates that monitoring progress toward individuals’ treatment goals is an appropriate method of evaluating the treatment effect of vortioxetine and overall improvement in patients with MDD. In particular, goal scores for each of the 3 goals set by each individual as part of the GAS-D approach have been found to be significantly correlated with the measures of depressive symptoms, QoL, and clinician-rated illness severity and improvement in patients administered vortioxetine [23]. Furthermore, improved function has been found to be significantly correlated with achieving self-defined goals [23].

The GAS-D is an important addition to the battery of clinical measurements used to assess the effect of antidepressant therapies. Patients and their healthcare providers have expressed a desire to move beyond the neurobiological management of depression to address the day-to-day functional impact of MDD [27]. Improvements in function, such as the decrease in work absences and increased work productivity observed at 12 weeks in patients treated with vortioxetine in this study, may be more important to patients than resolution of emotional symptoms alone [23]. For example, patients with MDD most commonly list improving family and other social relationships, increasing positive health behaviors, finding employment, and organizing their homes as desirable goals during treatment, in addition to relieving other depressive symptoms [28–30].

The goal attainment approach using the GAS-D also offers an effective method for assessing changes in specific functional domains, which may be overlooked by current standard measures that generally assess response to treatment by evaluating global functioning and symptomatic outcomes [28]. Indeed, functional outcomes are traditionally less responsive to treatment than symptomatic outcomes [30], and thus it is essential that specific functional outcomes important to patients are not overlooked. Approximately 40% of patients in this study achieved remission on standard outcome measures (PHQ-9 and CGI-S), yet 57.8% achieved a GAS-D score ≥ 50 at week 12. This finding suggests an apparent disconnect between measures of treatment success based on standardized clinical scales and outcomes considered to be meaningful for individual patients. Combining specific functional outcomes, such as emotional and physical goals, with global symptom outcome measures may provide a more comprehensive picture of treatment response, overall patient health, and QoL. Accordingly, investigators recommend moving toward the development and incorporation of functional, patient-centered outcome measures in clinical studies of antidepressant therapies [28].

### Limitations

This study has several limitations: First, real-world application of the goal attainment approach using the GAS-D outside of a formal clinical trial setting may require educating practitioners on how to use this tool to appropriately incorporate the GAS-D approach into their practices. Second, the scope of this study was limited to assessing the impact of GAS-D outcomes in patients switching to vortioxetine after receiving prior therapy. This single-arm study did not compare outcomes with vortioxetine versus placebo or other antidepressants with different modes of action, given that the study was designed as a preliminary investigation into the use of the GAS-D approach instead of a comparative study of treatment efficacy, which would have required a greater sample size to achieve sufficient statistical power. Any drug-related improvement in GAS-D score versus placebo would need to be assessed as part of a randomized controlled trial [13]. Third, there is preliminary evidence for the validity of GAS-D based on the post hoc convergent data presented in this study; however, more studies are needed.

## Conclusion

The GAS-D is a new instrument for assessing outcomes in patients with MDD that is complementary to current symptoms scales, such as the PHQ-9. The GAS-D provides a quantifiable framework for measuring progress against qualitative and diverse goals, enabling comparisons of qualitative outcomes between individual patients with MDD. Most importantly, this framework places the patient at the center of clinical outcome assessment by measuring treatment effectiveness in terms of whether treatment helped individual patients achieve their desired outcomes. This approach and framework align with goals supported by the National Committee for Quality Assurance and the Innovation and Value Initiative. Both organizations are exploring better ways to support and guide person-centered care to facilitate a shared understanding with providers and care teams of the individual’s goals and preferences to improve outcomes [31, 32]. The GAS approach has been shown to provide a complementary and clinically meaningful assessment applicable to a wide variety of disease states characterized by high interpatient variability and the need for individualized treatment plans, such as hemophilia and schizophrenia [33, 34]. To address the limitation noted for real-world application in MDD, research to develop mobile apps and electronic medical record platforms is underway to facilitate assessment of progress toward goal attainment [35, 36]. Accordingly, the GAS-D framework offers a scientifically valid and patient-centric endpoint that can be utilized in future studies to assess the effectiveness of antidepressants, evaluate functional improvements, and help better understand what matters most to patients during their treatment journey.

## Supplementary Information

**Additional file 1: Supplementary Figure 1.** GAS-D score formula. **Supplementary Figure 2.** Change in overall Q-LES-Q scores from baseline. **Supplementary Figure 3.** Change in LEAPS score from baseline.

**Additional file 2: Supplementary Table 1.** Correlations between goal scores and depressive symptoms, illness severity, and improvement [23]. **Supplementary Table 2.** Correlations between goal scores and Quality of Life (Q-LES-Q) [23]. **Supplementary Table 3.** Correlations between goal scores and cognitive performance/perceived deficit [23].

## Data Availability

The datasets generated and/or analyzed during the current study are available from the corresponding author upon reasonable request. The Goal Attainment Scale adapted for depression (Goal Attainment Scale – Depression © 2021 Takeda Pharmaceuticals U.S.A., Inc. All rights reserved) is available from Takeda Pharmaceuticals U.S.A., Inc., upon request.

## Abbreviations

AE: Adverse event
BMI: Body mass index
CBT: Cognitive behavioral therapy
CGI-I: Clinician Global Impression-Improvement
CGI-S: Clinician Global Impression-Severity
C-SSRS: Columbia-Suicide Severity Rating Scale
DSST: Digit Symbol Substitution Test
GAS-D: Goal Attainment Scale adapted for depression
ICH: International Council for Harmonisation of Technical Requirements for Pharmaceuticals for Human Use
IRB: Institutional review board
LEAPS: Lam Employment Absence and Productivity Scale
MDD: Major depressive disorder
NDRI: Norepinephrine-dopamine reuptake inhibitor
Q-LES-Q: Quality of Life Enjoyment and Satisfaction Questionnaire
PDQ-D: Perceived Deficits Questionnaire-Depression
PHQ-9: Patient Health Questionnaire-Depressive Symptoms
QoL: Quality of life
SD: Standard deviation
SMART: Specific, measurable, attainable, relevant, and time-bound
SNRI: Serotonin and norepinephrine reuptake inhibitor
SSRI: Selective serotonin reuptake inhibitor
TEAE: Treatment-emergent adverse event
VRFCAT: Virtual Reality Functional Capacity Assessment Tool
WHO-5: World Health Organization-Five Well-Being Index
