# Correction to: Using the Goal Attainment Scale adapted for depression to better understand treatment outcomes in patients with major depressive disorder switching to vortioxetine: a phase 4, single-arm, open-label, multicenter study

**DOI:** 10.1186/s12888-022-03798-2

**Published:** 2022-03-07

**Authors:** Maggie McCue, Sara Sarkey, Anna Eramo, Clement François, Sagar V. Parikh

**Affiliations:** 1 Takeda Pharmaceuticals U.S.A., Inc., 95 Hayden Avenue, Lexington, MA 02421 USA; 2 Lundbeck LLC, 6 Parkway North Blvd, Deerfeld, IL 60015 USA; 3 University of Michigan Health, 1500 E. Medical Center Dr, Ann Arbor, MI 48109 USA

**Correction to: BMC Psychiatry 21, 622 (2021)**

**http://orcid.org/10.1186/s12888-021-03608-1**

Following publication of the original article [1], the authors identified errors in the text. Below is a table of corrections which have been implemented in the original article.

**Table:** 

Section	Originally published text	Corrected text
Abstract	Treatment response and remission rates were 65 and 40%, respectively.	Treatment response and remission rates were approximately 65 and 40%, respectively.
Results - Baseline characteristics	Overall, study participants were predominantly white (69.2%), female (82.8%), and≤55years of age (77.9%). Mean PHQ-9 score was 15.7 (moderately severe depression), and 59.5% of the patients were employed.	Overall, study participants were predominantly white (69.2%), female (82.8%), and ≤55 years of age (77.9%). Mean PHQ-9 score was 15.7 (moderately severe depression), and 58.3% of the patients were employed.
Results - Safety and tolerability	Overall, 117 AEs were reported by 59 patients.	Overall, 117 AEs deemed related to study treatment were reported by 59 patients.
Availability of Data and Materials	The Goal Attainment Scale adapted for depression (Global Attainment Scale – Depression © 2021 Takeda Pharmaceuticals U.S.A., Inc. All rights reserved)	The Goal Attainment Scale adapted for depression (Goal Attainment Scale – Depression © 2021 Takeda Pharmaceuticals U.S.A., Inc. All rights reserved)

The original article [1] has been corrected.
