# Correction: Using the Goal Attainment Scale adapted for depression to better understand treatment outcomes in patients with major depressive disorder switching to vortioxetine: a phase 4, single-arm, open-label, multicenter study

**DOI:** 10.1186/s12888-022-03975-3

**Published:** 2022-06-08

**Authors:** Maggie McCue, Sara Sarkey, Anna Eramo, Clement François, Sagar V. Parikh

**Affiliations:** 1 Takeda Pharmaceuticals U.S.A., Inc., 95 Hayden Avenue, Lexington, MA 02421 USA; 2 Lundbeck LLC, 6 Parkway North Blvd, Deerfeld, IL 60015 USA; 3 University of Michigan Health, 1500 E. Medical Center Dr, Ann Arbor, MI 48109 USA

## Abstract

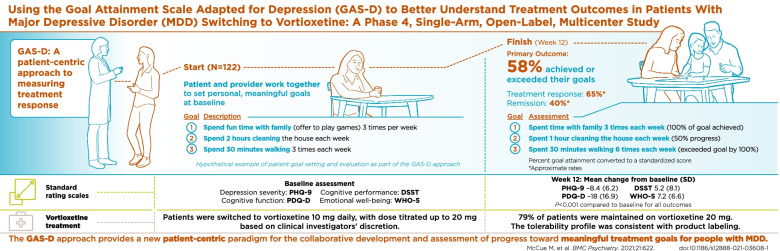

**Correction to: BMC Psychiatry 21, 622 (2021)**

**https://doi.org/10.1186/s12888-021-03608-1**

Following the publication of the original article [1], the authors would like to add the graphical abstract. The previous amendment can be found on 10.1186/s12888-022-03798-2.

The original article [1] has been corrected.
