# Supplementary material for: Using the Goal Attainment Scale adapted for depression to better understand treatment outcomes in patients with major depressive disorder switching to vortioxetine: a phase 4, single-arm, open-label, multicenter study
Source: BMC Psychiatry. 2021 Dec 11;21:622. doi: 10.1186/s12888-021-03608-1 (PMC8665619; doi:10.1186/s12888-021-03608-1)
Supplement: Supplementary file 1 — Additional file 1: Supplementary Figure 1. GAS-D score formula. Supplementary Figure 2. Change in overall Q-LES-Q scores from baseline. Supplementary Figure 3. Change in LEAPS score from baseline. [file 12888_2021_3608_MOESM1_ESM.pdf]

SUPPLEMENTARY MATERIAL

Supplementary Figure 1. GAS-D score formula

The formula to calculate overall GAS-D score was as follows:

$$\text{GAS Score} = 50 + \frac{10 * \sum (w_i x_i)}{\sqrt{0.7 * \sum w_i^2 +}\; 0.3 * (\sum w_i^2)}$$

w_i, goal weight; x_i, numerical outcome for individual goal

Abbreviation: GAS-D, Goal Attainment Scale Adapted for Depression.

Supplementary Figure 2. Change in overall Q-LES-Q scores from baseline

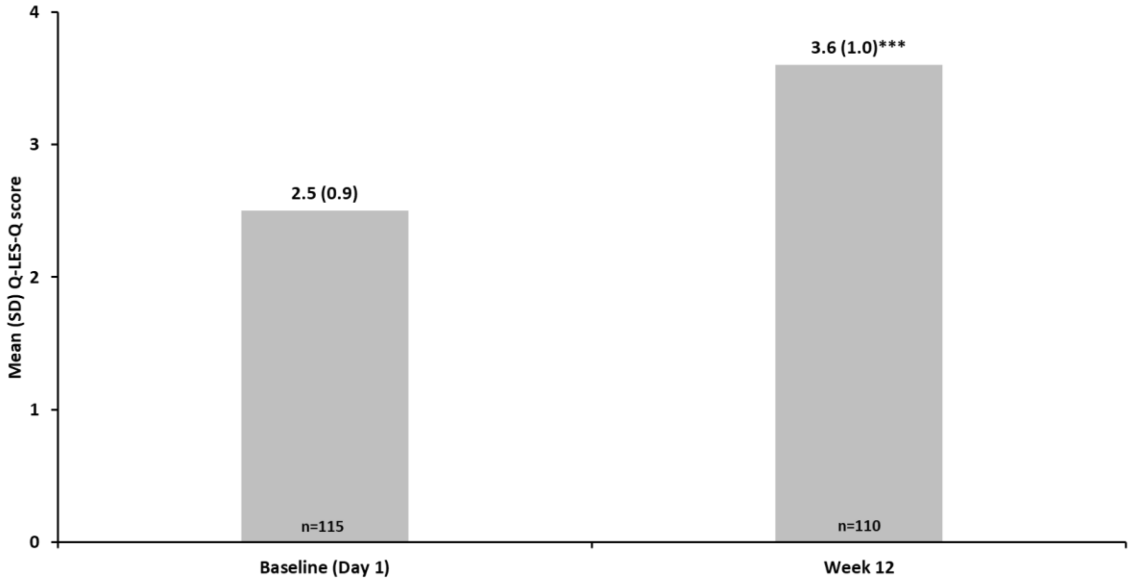

****P*<0.001 for change from baseline

Abbreviations: Q-LES-Q, Quality of Life Enjoyment and Satisfaction Questionnaire; SD, standard deviation.

Supplementary Figure 3. Change in LEAPS score from baseline

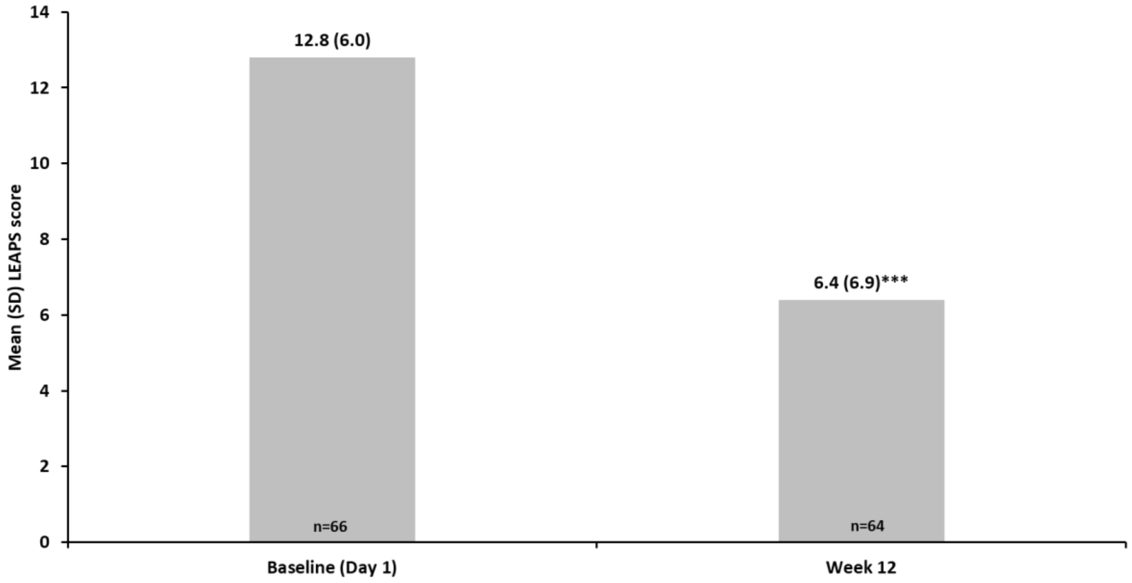

****P*<0.001 for change from baseline

Abbreviations: LEAPS, Lam Employment Absence and Productivity Scale; SD, standard deviation.
