# Supplementary material for: Using the Goal Attainment Scale adapted for depression to better understand treatment outcomes in patients with major depressive disorder switching to vortioxetine: a phase 4, single-arm, open-label, multicenter study
Source: BMC Psychiatry. 2021 Dec 11;21:622. doi: 10.1186/s12888-021-03608-1 (PMC8665619; doi:10.1186/s12888-021-03608-1)
Supplement: Supplementary file 2 — Additional file 2: Supplementary Table 1. Correlations between goal scores and depressive symptoms, illness severity, and improvement [23]. Supplementary Table 2. Correlations between goal scores and Quality of Life (Q-LES-Q) [23]. Supplementary Table 3. Correlations between goal scores and cognitive performance/perceived deficit [23]. [file 12888_2021_3608_MOESM2_ESM.pdf]

SUPPLEMENTARY MATERIAL

Supplementary Table 1. Correlations between goal scores and depressive symptoms, illness severity, and improvement [23]

**Convergent validity**			
	**PHQ-9**	**CGI-S**	**CGI-I**
**Baseline**			
**Goal 1 (naturalistic)**	0.063	0.086	–
**Goal 2 (from predefined domain)**	–0.072	0.214**	–
**Goal 3 (from predefined domain)**	0.01	–0.015	–
**Week 6**			
**Goal 1**	–0.198*	–0.217*	–0.267**
**Goal 2**	–0.220**	–0.204**	–0.267**
**Goal 3**	–0.368***	–0.303***	–0.277***
**Week 12**			
**Goal 1**	–0.381***	–0.303***	–0.364***
**Goal 2**	–0.333***	–0.392***	–0.385***
**Goal 3**	–0.334***	–0.353***	–0.358***
**Change over time**			
	**ΔPHQ-9[a]**	**ΔCGI-S[a]**	**ΔCGI-I[b]**
**ΔGoal 1**	–0.307***	–0.327***	–0.279*
**ΔGoal 2**	–0.413***	–0.240**	–0.243*
**ΔGoal 3**	–0.453***	–0.371***	–0.158

**P*<0.05 for correlation between goal score and clinical measures; ***P*<0.01 for correlation between goal score and clinical measures; ****P*<0.001 for correlation between goal score and clinical measures.

[a]Baseline to Week 12; [b]Week 6 to Week 12

Abbreviations: CGI-I, Clinical Global Impression Scale-Improvement; CGI-S, Clinical Global Impression Scale-Severity; PHQ-9, Patient Health Questionnaire-Depressive Symptoms.

Supplementary Table 2. Correlations between goal scores and Quality of Life (Q-LES-Q) [23]

**Convergent validity**				
	**Q-LES-Q domain**			
	**Physical**	**Social**	**Work**	**Feeling**
**Baseline**				
**Goal 1 (naturalistic)**	0.081	0.203*	0.063	0.069
**Goal 2 (from predefined domain)**	0.081	–0.014	0.028	0.095
**Goal 3 (from predefined domain)**	0.028	–0.089	0.018	0.135
**Week 12**				
**Goal 1**	0.271***	0.368***	0.382***	0.286**
**Goal 2**	0.35***	0.323***	0.222*	0.346***
**Goal 3**	0.313**	0.317**	0.317**	0.257**
**ΔGAS and ΔQ-LES-Q change over time**				
**ΔGoal 1**	0.25**	0.272**	0.322***	0.387***
**ΔGoal 2**	0.32***	0.204*	0.203	0.291**
**ΔGoal 3**	0.309***	0.198*	0.248*	0.376***
**Baseline GAS score and ΔQ-LES-Q change over time**				
**Goal 1**	–0.058	–0.086	–0.029	–0.07
**Goal 2**	–0.103	–0.038	–0.229*	–0.11
**Goal 3**	0.054	0.128	0.093	0.025

*P<0.05 for correlation between goal and Q-LES-Q scores; **P<0.01 for correlation between goal and Q-LES-Q scores; ***P<0.001 for correlation between goal and Q-LES-Q scores.

Abbreviations: GAS, Goal Attainment Scale; Q-LES-Q, Quality of Life Enjoyment and Satisfaction Scale.

Supplementary Table 3. Correlations between goal scores and cognitive performance/perceived deficit. [23]

**Convergent validity**			
	**DSST**	**PDQ-D5**	**PDQ-D**
**Baseline**			
**Goal 1 (naturalistic)**	0.024	–0.177*	–0.18*
**Goal 2 (from predefined domain)**	–0.021	0.081	0.046
**Goal 3 (from predefined domain)**	0.059	0.019	0.018
**Week 6**			
**Goal 1**	0.107	–0.201*	–0.225***
**Goal 2**	0.079	–0.11	–0.081
**Goal 3**	0.013	–0.127	–0.142*
**Week 12**			
**Goal 1**	0.332**	–0.251*	–0.249*
**Goal 2**	0.064	–0.267**	–0.233*
**Goal 3**	0.197*	–0.312**	–0.311**
**∆GAS and ∆DSST/PDQ-D5/PDQ-D change over time**			
**∆Goal 1**	0.201*	–0.356**	–0.338**
**∆Goal 2**	0.125	–0.315**	–0.352**
**∆Goal 3**	0.023	–0.503**	–0.488**
**Baseline GAS score and ∆DSST/PDQ-D5/PDQ-D change over time**			
**Goal 1**	–0.038	0.04	0.036
**Goal 2**	–0.065	–0.027	–0.018
**Goal 3**	–0.107	0.01	0.022

*P<0.05 between goal score and measure; **P<0.01 between goal score and measure; ***P<0.001 between goal score and measure.

Abbreviations: DSST, Digit Symbol Substitution Test; GAS, Goal Attainment Scale; PDQ-D, Perceived Deficit Questionnaire-Depression; PDQ-D5, 5-item Perceived Deficit Questionnaire-Depression.
